# Subcutaneous nephrovesical bypass: Treatment for ureteral obstruction in advanced metastatic disease

**DOI:** 10.3892/ol.2014.2679

**Published:** 2014-11-06

**Authors:** YUNYAN WANG, GONGCHENG WANG, PEIJIN HOU, HAIJUN ZHUANG, XIAOSONG YANG, SHUO GU, HENGBING WANG, LU JI, ZONGYUAN XU, JUNSONG MENG

**Affiliations:** Department of Urology, Huai’an First People’s Hospital, Nanjing Medical University, Huai’an, Jiangsu 223300, P.R. China

**Keywords:** subcutaneous nephrovesical bypass, ureteral obstruction, malignant disease

## Abstract

The aim of the present study was to explore the value of subcutaneous nephrovesical bypass (SNVB) for the treatment of ureteral obstruction due to pelvic metastatic disease. SNVB stents (n=30) were implanted in 24 patients with advanced metastatic disease between January 2008 and December 2012. Urinalysis, serum creatinine (SCr), glomerular filtration rate (GFR), quality of life (QoL) scores, and renal ultrasonography were evaluated at follow-up. The SNVB procedures were successful in all 24 patients. Patient follow-ups occurred at an average of 10.6 months. Preoperative hydronephrosis was eliminated in 16 cases (53.3%) and reduced in the remaining patients. Following surgery, SCr levels reduced significantly from 256±46 to 124±23 μmol/l (P<0.001). GFRs increased from 25±4.8 to 45±5.3 ml/min (P<0.01). The mean QoL scores were 3.4±1.4 preoperatively and 7.6±1.0 postoperatively (P<0.001). The results showed that SNVB is a minimally invasive, effective and safe procedure for patients with ureteral obstruction resulting from advanced malignant disease. As an alternative procedure to percutaneous nephrostomy, SNVB offers patients a better QoL.

## Introduction

A number of patients experience unilateral or bilateral hydronephroses due to advanced pelvic tumor compression, ureteral malignant invasion or retroperitoneal fibrosis caused by radiation therapy. Subcutaneous nephrovesical bypass (SNVB) is a minimally invasive, effective and safe procedure for patients with ureteral obstruction resulting from advanced malignant disease. As an alternative procedure to percutaneous nephrostomy, SNVB offers patients a better quality of life (QoL) (1). Retrograde insertion of a double J stent is the first-line therapy; however, stent failure occurs in nearly half of all treated patients (2). An alternative option is to perform a permanent percutaneous nephrostomy (PCN), which has become a safe and widely used technique in the last 20 years (3,4). However, PCN diminishes patient QoL due to a number of possible complications, including obstruction or dislocation of the nephrostomy tube and urinary tract infection (UTI) (5). The aim of the present study was to maintain an acceptable patient QoL and restore kidney function by performing SNVB in 24 patients.

## Materials and methods

### Patients

SNVB stents (N=30) were implanted in 24 patients diagnosed with unilateral or bilateral obstructed ureters due to progressive metastatic, end-stage disease at the Department of Urology, Huai’an First People’s Hospital, Nanjing Medical University (Huai’an, China) between January 2008 and December 2012. Patients included 14 males and 10 females with a mean age of 56.6 years (range 42–73 years). The cases either displayed bilateral ureteral obstruction (n=6), a left ureteral obstruction (n=11) or a right ureteral obstruction (n=7). Among the 24 patients, six had colorectal carcinoma, five had esophageal cancer, five had uterine cervical cancers, four had gastric carcinoma, two had ovarian cancers and two had prostatic adenocarcinoma. Patients with radiation cystitis were excluded. All of the selected patients provided written informed consent prior to entering this study, and the study was approved by the ethics committee of Huai’an First People’s Hospital, Nanjing Medical University, (Huai’an, China).

All patients had advanced pelvic malignancy compression or invasion resulting in unilateral or bilateral ureteral obstruction as observed by magnetic resonance imaging (MRI) and retrograde insertion. Double J stenting was impossible for all patients studied, and 10 patients had been treated previously with PCN. Urinalysis, serum creatinine (SCr), glomerular filtration rate (GFR) and ultrasonography were measured in all patients preoperatively, 3 days postoperatively, and every 3 months after that at follow-up appointments, and patient QoL scores were evaluated. All patients were monitored at follow-up appointments until they succumbed to malignant disease.

### Operative technique

An SNVB set consists of a 9F/54-cm special double J stent as a nephrovesical bypass, an 18-G renal puncture needle, a guide wire, an 8–12 F fascia dilator, a 12 F/35-cm malleable tunneler and a 12F half-trough bladder puncture needle (C.R. Bard, Inc., Murray Hill, NJ, USA). Each tip of the J stent is open and the side holes only appear within the curved part.

All patient procedures were performed under general anesthesia by the same surgeon. The first eight patients were initially placed in a prone position for kidney access, and then rotated to an anterior oblique elevation (45°) position to access the bladder and place the distal part of the bypass. As the procedures were completed, it was determined that a more effective approach would be to place subsequent patients in an anterior oblique elevation (45°) position immediately to permit access to the kidney and bladder simultaneously without having to change the patient position.

Under ultrasound guidance, needle puncture to the inferior calyceal system was performed from the posterior axillary line. The distance was measured from the skin to the target renal calyx by ultrasound (Pro Focus 2202; BK Medical Ultrasound Systems, Denmark). The guide wire was placed into the target renal calyx when urine was detected in the needle. Tracts were dilated to 12F using sequential Amplatz dilators (C.R. Bard, Inc., Murray Hill, NJ, USA). The pelvic component of the bypass tube was placed into the renal pelvis along the guide wire. The depth of the inserted stent was in accordance with the distance from the skin to the target renal calyx, excluding the curved part, so that the curved part was located in the target renal calyx. The stent was fixed within subcutaneous tissue with a 3-0 nylon suture to prevent stent dislocation. The skin entry site was widened by ~1 cm and the subcutaneous tunnel was extended from the nephrostomy incision to the point 2–3 cm above the pubic symphysis using the 12F malleable tunneler. A 1-cm incision was created where the tunneler punctured the suprapubic skin. The pendulous section of the stent was inserted into the hollow tunneler from the nephrostomy incision to the suprapubic incision. The stent was fixed by hand and the tunneler was extracted from the suprapubic incision. Via the small skin incision, a cystostomy was established using a 12F half-trough bladder puncture needle under direct vision of flexible cystoscopy. The bladder component of the bypass stent was inserted into the bladder under wire guidance. The stent was fixed to the subcutaneous tissue. The two small skin incisions were closed with 3-0 absorbable sutures. A urethral catheter was inserted for 1 week. Antibiotic prophylactics were administered 24 h preoperatively and 5 days postoperatively.

### Statistics

To compare numerical variables, a two-paired Student’s t-test was used, and P<0.05 was considered to indicate a statistically significant difference (Statistical Analysis Software, V8.0; SAS Institute Inc., Cary, NC, USA).

## Results

**Figure 1:**
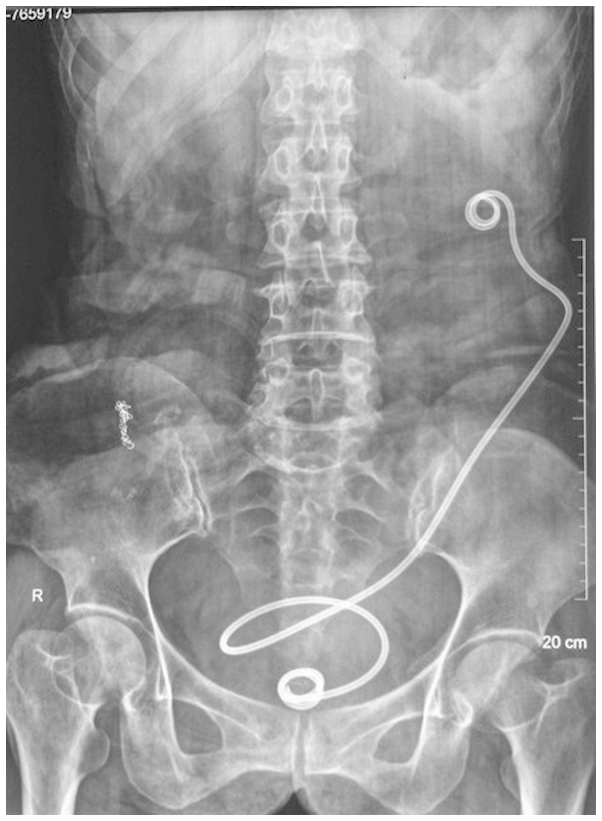
A representative kidney, ureter and bladder X-ray film following the subcutaneous nephrovesical bypass (SNVB) procedure. The SNVB bypass was implanted for a left ureteral obstruction and an appropriate site was chosen. The upper curved part of the bypass is located in left kidney, with the lower part in the bladder.

In total, 30 SNVB stents were successfully implanted in 24 patients. No operative or immediate postoperative mortalities occurred. The mean operation time was 78 min (range, 52–118 min) for SNVB. A kidney, ureter and bladder radiology film captured 3 days post-surgery confirmed that the SNVB stents were properly placed (Fig. 1).

Mean patient follow-up time was 10.6 months (range, 6–36 months). The SNVB stents were well tolerated, but during follow-up, all but six patients succumbed to progressive metastatic disease. Following the surgical procedure, hydronephrosis was completely resolved in 16 of 30 affected kidneys (53.3%) and was reduced in the remaining kidneys. Patient data for preoperative and postoperative outcomes are provided in Table I. There were no major perioperative complications. Certain patients experienced mild hematuria, which disappeared after 1–2 days. Common procedural complications are presented in Table II.

## Discussion

Malignant pelvic tumors or advanced metastasis often results in unilateral or bilateral ureteral obstruction (6), and this can lead to nephrosis, renal insufficiency and uremia. These problems may be solved with retrograde ureteric stenting, which requires periodic stent changes (7). Gradually, retrograde insertion of a double J stent in the ureter may fail in the presence of advanced pelvic malignancies, or it may be complicated by infection or obstruction (8). Alternatively, a PCN may be used; however, this requires an external urine collection device (9). As the malignancy progresses, patients not only suffer pain due to the advanced tumors, but also experience nephrostomy complications, including infection, obstruction and slippage of the nephrostomy tube (10). Additionally, patients experience a number of inconveniences, including nephrostomy tube and urine bag changes, bathing with the nephrostomy tube and social issues, all of which compromise QoL (11,12).

SNVB is not affected by the ipsilateral ureter and it offers minimal invasion, low risk and easy manipulation (13). Research shows that no severe complications occur during this operation, except for the occasional urinary extravasation and local infection (14). In the current study, all patients underwent successful surgery and experienced no severe complications. In the postoperative follow-up period, no bypass stent displacement or stone formation occurred. Ipsilateral hydronephrosis and GFR improved markedly. SCr levels were close to normal and remained stable, and the uremic patient no longer required dialysis. Patients expressed no typical complaints, such as back pain and urinary symptoms. QoL scores increased postoperatively and these data are in accordance with previous findings (15). Compared with PCN patients, the patients of the current study no longer required external urine collection bags or associated equipment, and SNVB offered greater comfort for sleeping and improved mobility compared with conventional PCN (16).

SNVB is suitable for patients with ureteral obstruction due to advanced abdominal pelvic malignancy without radical surgery. Patients receiving the SNVB procedure should have a functional bladder and their disease must exclude lower urinary tract symptoms. SNVB can replace permanent PCN when a double J stent is not able to be inserted into the ureter endoscopically (17,18). In the present study, 14 patients were offered SNVB as end-stage malignancies had reduced their life expectancy to <12 months.

The results of the current study indicate that the single double J stent used in the procedure was superior to two J-tubes connected at the midpoint by a metal connector, which may result in potential urinary extravasation (16,17). SNVB stents made of different materials have been reported to withstand implantation for 6–84 months (19). Regular changing of the SNVB is not necessary, and long-term complications were rare even in diabetic patients. SNVBs may be replaced in the face of complications if patient survival times permit this. Changing the SNVB is not difficult: The existing subcutaneous channel for the long-term indwelling bypass stent aids in the replacement (20). Additionally, PCN may be performed even if the SNVB could not be successfully replaced.

The surgery requires attention to be paid to perioperative events. Primarily, UTI is common in patients with ureteral obstructions, particularly in end-stage patients with malignant diseases. Infection must be treated with antibiotics to prevent severe complications and procedure failure. Additionally, the puncture point for renal puncture should be selected in the posterior axillary line to prevent puncturing the peritoneal cavity and damaging the bowel. In certain PCN procedures, a new nephrostomy would be established and isolated from the existing nephrostomy to reduce infection. The lower calyx should be chosen as the target renal calyx to facilitate urine drainage. Finally, the depth of bypass placement in the renal pelvis should be measured by ultrasound and be between the puncture point and the target renal calyx. The curved tips with side holes of the bypass should be placed within the renal pelvis and bladder, preventing postoperative urinary extravasation. Infection and bypass obstruction should be prevented to prolong the bypass stent retention time. For instance, patients should drink water and urinate as needed, specifically voiding again following an initial urination to eliminate urine reflux by the bypass stent.

In conclusion, SNVB is a minimally invasive, safe and effective procedure that can improve renal pelvic drainage for ureteral obstruction patients with end-stage malignancies. SNVB offers patients a better QoL and should be considered an alternative procedure to PCN, which is documented to reduce QoL due to the need for cumbersome external urine collection devices.

## Figures and Tables

**Table I:** GFR, serum creatinine, QoL mean value before and after operation (range).

	Glomerular filtration rate (ml/min)	Serum Creatinine (μmol/l)	QoL
Pre operation	25±4.8 (18–26)	256±46 (85–662)	3.4±1.4 (0–5)
Post operation	45±5.3 (28–58)	124±23 (88–176)	7.6±1.0 (5–9)
P-value	<0.01	<0.001	<0.001

GFR, glomerular filtration rate; QoL, quality of life.

**Table II:** Complications related to the procedure.

Complication	n (%)
Mild hematuria	10 (33.3)
Urinary tract infection	6 (23.1)
Urinary urgency	5 (16.7)
Subcutaneous infection	2 (6.7)

The respective percentages refer to 30 stents placed in 24 patients.
